# Insights on Mediterranean Diet from the SUN Cohort: Cardiovascular and Cognitive Health

**DOI:** 10.3390/nu12051332

**Published:** 2020-05-08

**Authors:** Justyna Godos, Fabio Galvano

**Affiliations:** 1 Oasi Research Institute—IRCCS, 94018 Troina, Italy; 2 Department of Biomedical and Biotechnological Sciences, University of Catania, 95123 Catania, Italy; fgalvano@unict.it

**Keywords:** Mediterranean diet, SUN cohort, risk factor, mortality, cardiovascular, cognitive, mental health, depression, quality of life

## Abstract

Epidemiological evidence has demonstrated the association between dietary factors and non-communicable diseases. Great attention has been payed to the Mediterranean dietary pattern, given its richness in anti-oxidant and anti-inflammatory molecules. Numerous reports from the SUN (Seguimiento Universidad De Navarra) cohort have shown that higher adherence to the Mediterranean dietary pattern may be inversely associated with mortality, type 2 diabetes and cardiovascular diseases. Likewise, a link with mental health outcomes, such as depressive symptoms, cognitive status and quality of life was also found, suggesting its beneficial effects toward brain health.

Non-communicable diseases are the leading causes of premature mortality and disability in developed countries [1,2]. Obesity rates are growing worldwide [3], representing an important metabolic risk factor for non-communicable diseases [4]. Among various behavioural risk factors, dietary risks account for 11 million deaths and 255 million disability-adjusted life years [5]. Based on current international guidelines, causal inference in nutrition science is supported when agreement between intervention and observational studies occurs [6]. Up to today, great efforts have been made to assess the level of evidence for the association between exposure to dietary factors and the risk of non-communicable diseases [7,8,9,10,11,12], and results suggest that several components characteristic of the Mediterranean dietary pattern may play a role in affecting human health.

The SUN (Seguimiento Universidad De Navarra) study represents one of the main cohorts in the European Mediterranean area aiming to explore the association between dietary factors and non-communicable diseases. It involves individuals aged 20 years and over who have previously graduated from the University of Navarra, as well as from other different Spanish universities; the cohort has adopted an open continuous recruitment of highly educated participants, which is supposed to reduce the potential risk of bias. The project has contributed for over 20 years to evidence on the importance of traditional dietary patterns, such as the Mediterranean diet, as an ideal dietary choice for the prevention of chronic diseases [13]. The article published by Carlos et al. [14] provided an overview of the main findings from the studies published within the context of the SUN cohort: the study showed that better adherence to the Mediterranean diet was significantly associated with a lower risk of all-cause mortality [15], cardiovascular disease (CVD) incidence and mortality [16], and, more recently, a composite outcome including all-cause mortality, CVD and type-2 diabetes [17]. These results may be explained by several findings reported for cardiovascular risk factors, including a decrease in mean systolic and diastolic blood pressure [18], reduction in the risk of developing type-2 diabetes [19] and reduction in weight gain over the follow up period [20]. The recent Prevención con Dieta Mediterránea (PREDIMED) study [21] and supporting meta-analyses comprehensively assessing the evidence on the association between adherence to the Mediterranean diet and cardiovascular outcomes in prospective cohort studies [22,23] provided substantially comparable risk estimates of cardiovascular outcomes. Also, the findings on type-2 diabetes and cardiovascular risk factors have been included in meta-analyses of cohort studies [24] and replicated in an experimental setting, confirming the consistent healthy effects of higher adherence to the Mediterranean diet against blood glucose impairment, insulin sensitivity and type-2 diabetes risk [25].

Another line of research investigated within the context of the SUN cohort has been the role of the Mediterranean diet on mental health, including depressive symptoms, cognitive status and quality of life. Higher adherence to the Mediterranean and other healthy dietary patterns has been associated with a lower risk of depression [26]. Similarly, individuals with low and moderate adherence to the Mediterranean diet showed lower scores in cognitive function than subjects with a higher adherence [27]. These results have been consistent with the investigation on quality of life in which higher adherence to the Mediterranean diet was associated with better self-perceived mental health (as a component of quality of life) [28]. The results from the comprehensive evaluation of observational studies showed no significant association between adherence to the Mediterranean diet and risk of depression in cohort studies, but an inverse significant association with odds of depression in cross-sectional studies was reported [29]. Findings on cognitive status from the SUN cohort confirm, at least in part, the results from meta-analyses on Mediterranean diet and cognitive function, which report a significant association between higher adherence to the Mediterranean diet and older adults’ episodic memory and global cognition, but not working memory or semantic memory [30]. Similarly, findings from clinical trials provided evidence of a reduced risk of dementia [31], but mostly non-significant results in terms of cognitive functioning and brain morphology [32].

A traditional Mediterranean diet characterized by (i) high intake of plant-derived foods (including fruit, vegetables and legumes); (ii) use of olive oil, nuts and fish as the main sources of fats; (iii) low consumption of meat and processed foods; and (iv) moderate alcohol intake (mostly wine) during meals, and is a dietary pattern that provides an ideal content of healthy nutrients (such as poly- and monounsaturated fatty acids) and bioactive components exerting anti-inflammatory and antioxidant properties (such as vitamins and polyphenols) [33], many of which have been reported to modulate molecular pathways and play a role in CVD [34] and mental disorder prevention [35]. Thus, conclusions regarding the prevention of cardiovascular diseases and cognitive decline are well supported, while further evidence is needed for other major outcomes.

The contribution of the SUN cohort to nutritional science was crucial in making modern knowledge on the role of certain dietary factors, among which the Mediterranean diet is the core research question of this cohort, in human health. The results obtained are in line with those reviewed in two umbrella reviews of meta-analyses of the role of the Mediterranean diet in preventing non-communicable diseases [36,37]. The data provided is valuable, as a recent overview of literature showed a global decrease in adherence to the Mediterranean diet [38,39], while some surveys based in countries traditionally characterized by this dietary pattern (i.e., Italy and Greece) showed a nutrition transition process and a slow desertion of a Mediterranean-type dietary pattern in favour of more “Westernized” diets, rich in (ultra)processed foods, trans-fatty acids, refined cereals and added sugars [40,41,42]. Lately, higher adherence to the Mediterranean dietary pattern has been also proven to be a sustainable choice for the environment, as reported by the SUN and other Mediterranean cohorts [43,44,45]. However, as for all other prospective cohort studies, the SUN cohort shares similar limitations that should be taken into account when considering its results: recall bias, over- or under-estimation of food intake, reverse causation and confounding variables due to the clustering of healthy/unhealthy lifestyle habits are common limitations that cannot be overcome with an observational study design. Nonetheless, the findings from the SUN cohort obtained up until now have been ground-breaking in leading the way toward a better understanding of the role of the Mediterranean diet on human health. The future application of modern epidemiological approaches investigating genetic variants, gut microbiota, big data measurement and analysis will help to disentangle aspects related to the Mediterranean diet.
